# Exposure Render: An Interactive Photo-Realistic Volume Rendering Framework

**DOI:** 10.1371/journal.pone.0038586

**Published:** 2012-07-02

**Authors:** Thomas Kroes, Frits H. Post, Charl P. Botha

**Affiliations:** 1 Department of Intelligent Systems, Delft University of Technology, Delft, The Netherlands; 2 Department of Orthopaedics, Leiden University Medical Centre, The Netherlands; 3 LKEB, Department of Radiology, Leiden University Medical Centre, The Netherlands; Institute of Psychology, Chinese Academy of Sciences, China

## Abstract

The field of volume visualization has undergone rapid development during the past years, both due to advances in suitable computing hardware and due to the increasing availability of large volume datasets. Recent work has focused on increasing the visual realism in Direct Volume Rendering (DVR) by integrating a number of visually plausible but often effect-specific rendering techniques, for instance modeling of light occlusion and depth of field. Besides yielding more attractive renderings, especially the more realistic lighting has a positive effect on perceptual tasks. Although these new rendering techniques yield impressive results, they exhibit limitations in terms of their exibility and their performance. Monte Carlo ray tracing (MCRT), coupled with physically based light transport, is the de-facto standard for synthesizing highly realistic images in the graphics domain, although usually not from volumetric data. Due to the stochastic sampling of MCRT algorithms, numerous effects can be achieved in a relatively straight-forward fashion. For this reason, we have developed a practical framework that applies MCRT techniques also to direct volume rendering (DVR). With this work, we demonstrate that a host of realistic effects, including physically based lighting, can be simulated in a generic and flexible fashion, leading to interactive DVR with improved realism. In the hope that this improved approach to DVR will see more use in practice, we have made available our framework under a permissive open source license.

## Introduction

Realistic illumination in volume visualization plays a central role in 3D shape perception. For example, the user study performed by Lindemann et al. [1], in which the effectiveness of seven state of the art DVR techniques is measured, clearly showed that global illumination models help in assessing depth and size in images. Furthermore, Ropinski et al. [2] developed a realistic lighting model for volume rendering, and demonstrated that by using realistic lighting, observers use less time and are more accurate at assessing depth in a volume rendering.

Recent years have seen a great deal of research towards enhancing interactive *Direct Volume Rendering* (DVR) approaches with more realistic illumination, for example ambient occlusion [3], shadows [4], [5], realistic scattering [6], [7] and global illumination [8]. However, research up to now has focused on fast approximations of illumination that could be integrated with GPU-based volume renderers, both texture- and raycasting-based, as the physically-based modeling of illumination was considered to be prohibitively expensive.

In contrast to many of the existing approximations, Monte Carlo ray tracing (MCRT), combined with physically based light transport, is able to simulate real-world light interaction without compromising accuracy of light transport computations, thus resulting in more realistic images. Monte Carlo rendering algorithms are capable of dealing with complex lighting, material and camera configurations. It has been demonstrated that MCRT, with suitable modifications addressing hardware peculiarities, can be performed on the the GPU [9], [10]. However, to the best of our knowledge, MCRT has not yet been applied to the complete interactive DVR pipeline in order to achieve photo-realism. The work done by Salama et al. [6] comes the closest, but is based on explicitly using isosurfaces in the rendered volume data. This distinction is discussed in more detail in the *Related Work* section.

In this work, we apply MCRT to the interactive rendering of volumetric datasets, sampling the whole domain and taking into account the full gamut of volume densities. In order to combine surface and volumetric scattering, we introduce *hybrid scattering*.

Our DVR framework is able to generate high quality images at interactive speeds. It builds up images progressively, where a recognizable rendering appears within a fraction of a second and image quality increases rapidly. The rendering can be interacted with from the very start. All scene parameters, e.g., transfer function, camera and lighting, can be modified interactively. Based on our experiments we conclude that stochastic MC based simulation of light transport is an attractive solution to the problem of photo-realistic rendering in interactive DVR. Stochastic MC based simulation of light transport is particularly interesting because it enables the integration of various physically based effects into a unified approach without significant effort, whereas other solutions restrict the number of lights, the shape of lights, the camera model, and so forth. Furthermore, due to its sampling approach, problems with aliasing and stepping artifacts are dealt with easily. The DVR framework is able to cope with complex lighting on the fly, and the increased quality of the images help to convey shape and detail.

With this work, our contributions are the following:

- We demonstrate that MCRT is an appealing approach to DVR, allowing a host of effects and flexible lighting schemes, and that it can be run efficiently on commodity graphics hardware using CUDA, with the necessary performance optimizations.
- We present a re-usable GPU-based interactive direct volume renderer that integrates stochastic ray-traced lighting, thus enabling physically-based volumetric shadows, any number of arbitrarily positioned, shaped and textured area lights and finally the modeling of a real-world camera, including its lens and aperture. Our complete implementation is available under a permissive open source license, hopefully stimulating collaboration and allowing others to reproduce and further improve our work.

**Figure 1:**
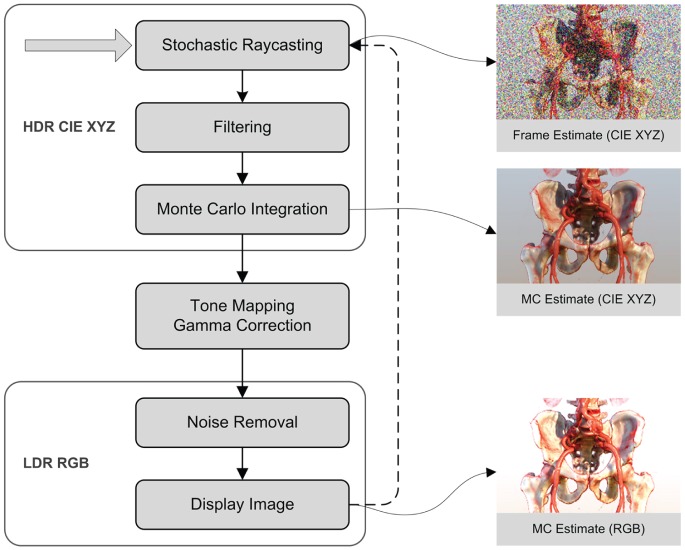
A high level overview of our rendering pipeline.

In 2009, Banks and Beason reported that the “”market penetration of physically-based illumination in scientific visualization was nearly zero in 2008, despite many indications in research that it has perceptual advantages [11]. One of the reasons they cited, was purely that the scientists generating the visualizations did not have easy access to global illumination implementations in their standard workflow. By making our work available as a reusable and permissively licensed implementation, we hope to contribute to the uptake of physically-based illumination in interactive direct volume rendering.

**Figure 2:**
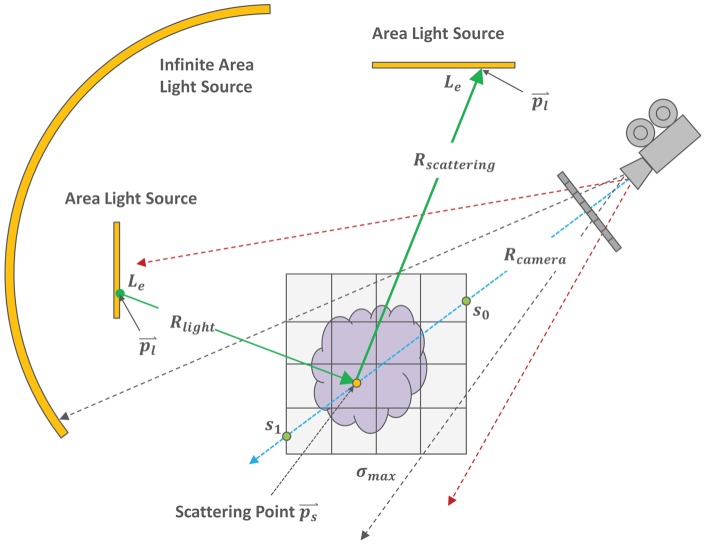
High level overview of stochastic ray-casting in our DVR framework. For every ray that is traversed, one scattering point is stochastically determined and the light contribution is computed using two additional rays.

**Figure 3:**
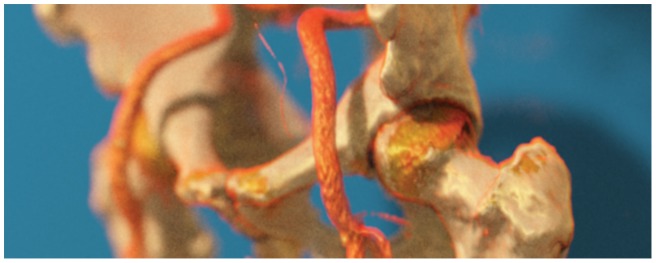
Depth-of-field rendering in Exposure Render.

The rest of the paper is structured as follows. In the *Related work* section we survey related work on volumetric shadows, ambient occlusion and physically-based light transport. In the *Method* section, we document our technique and in the *Results* section we analyze the performance and present some example renderings. Finally in the *Conclusions* section we summarize our findings and point out directions for future research.

### Related work

Global illumination, and especially shadows, are compelling ways of conveying depth and shape in 3D visualization in general [12] and in volume rendering specifically. Some of the first improvements of the more straightforward light transport approximations, such as that presented by Max [13], were made with the introduction of shadows in volume rendering. Behrens and Ratering introduced shadows in volume rendering by pre-computing a shadow volume, for a given relative light source position, that could then be rendered using a standard texture-based volume rendering algorithm [4]. Kniss et al. presented a volumetric lighting model that integrated half angle slicing, a texture-based volume rendering technique to calculate volumetric shadows, a lookup table-based phase function implementation and an approximation of multiple forward scattering based on aggregating light from previous slices [14], [15].

**Figure 4:**
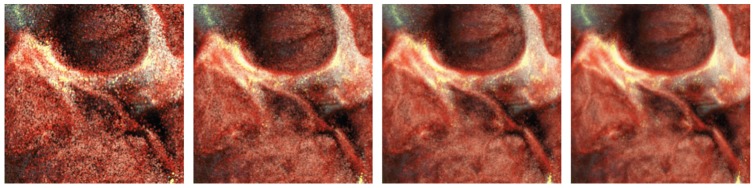
Image progression without noise reduction. The initial iterations show high sample variance.

Hadwiger et al. [5] adapted deep shadow maps [16], a technique for computing semi-transparent volumetric shadows, to raycasting on the GPU. Ropinski et al. [17] presented an alternative implementation of deep shadow maps for GPU raycasting that supported caching when the light source configuration was kept constant, and compared it to normal shadow maps (non-transparent shadows) and shadow rays. In all cases, volume rendering realism was greatly improved with the integration of shadows. However, the mentioned examples were all limited to modeling a single point light source. The DVR framework in this paper does not pose restrictions on the lighting configuration, e.g., the number of lights, their shape and finally their texturing.

**Figure 5:**
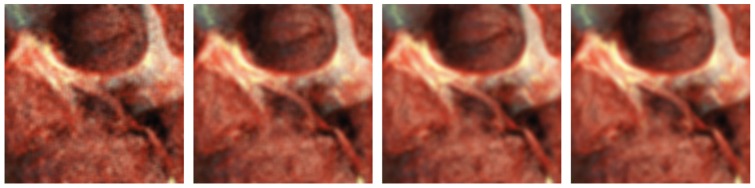
The same data set and configuration as in Figure 4, rendered with a noise reduction filter. By applying the noise reduction during the initial iterations, the objectionable noise at the startup of the MC algorithm is reduced to a great extent, at the expense of slightly increased blurring during the initial iterations. The influence of noise filtering, and thus the amount of blurring, reduces based on the error in the running estimate.

Ambient occlusion (AO), introduced by Zhukov et al. [3] with the term *obscurances*, is an effective and usually inexpensive technique for approximating global illumination. AO computes the light intensity on a shading point by determining the hemispherical occlusion of environmental light. In their survey, Méndez-Feliu and Sbert point out the difference between obscurances and ambient occlusion: Whilst the latter represents the degree of openness of a point, the former also takes into account diffuse indirect lighting, yielding more physically correct lighting and for example color bleeding effects [18]. However, the two terms are often used interchangeably. The vicinity shading method introduced by Stewart [19] was the first to incorporate ambient occlusion into volume rendering, with a method called vicinity shading. Their method uses neighboring voxels and their obscurances to compute local illumination, which results in darkened crevices and depressions. Their method requires preprocessing for every scene modification and requires an additional buffer to store the results of the illumination. Penner and Mitchell [20] used histograms to classify the obscurance around a voxel. The method by Ropinski et al. [7] used local histogram clustering for the pre-computation of occlusion information. It is important to note that AO and even obscurance do not take into account a specific light position, but are both based on the geometric occlusion of a sample point, and hence approximate the light that could conceivably reach that point. In the DVR framework presented in this paper, the entire volume domain is taken into account for shadow computations.

**Figure 6:**
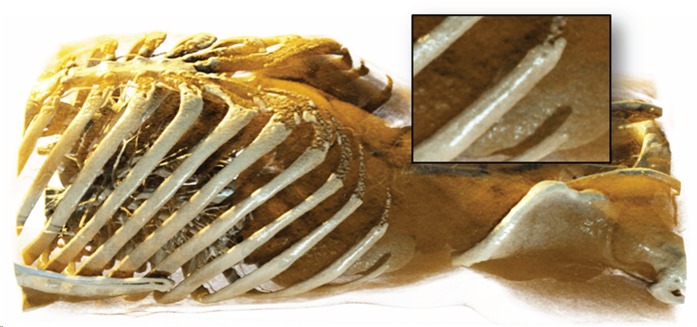
Image rendered with hybrid scattering. Note how detail is preserved through specular highlights, while still fully supporting volumetric scattering.

**Figure 7:**
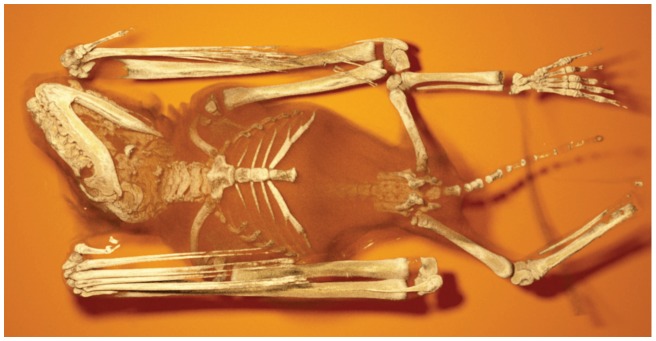
Volume rendering of a bat. Note the soft shadows on the plane behind the CT data caused by a planar area light.

**Figure 8:**
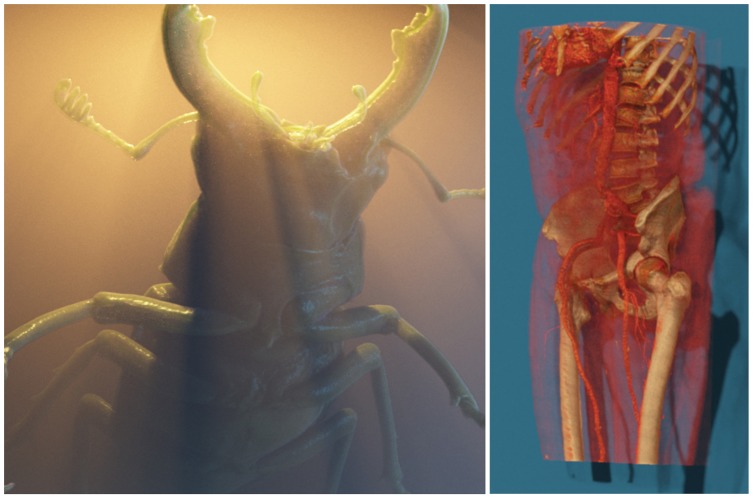
Left: Our method deals well with thin participating media. Right: Integration of physically based surface and volumetric scattering functions.

**Figure 9:**
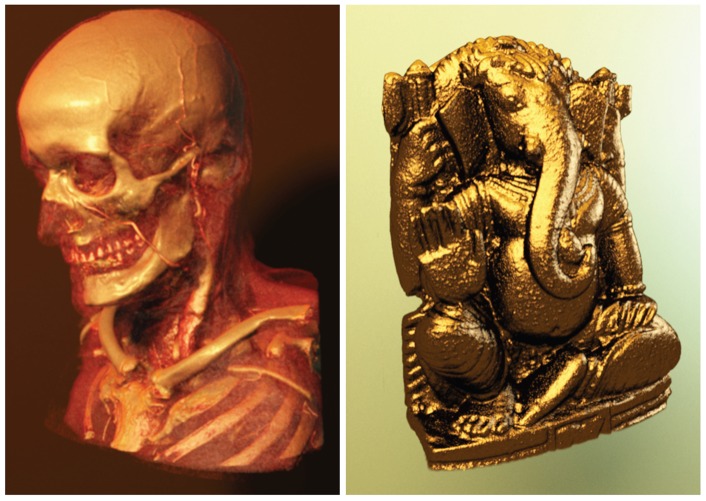
Left: Rendering of the publicly available Manix Data set (Osirix) with a photographic lighting setup. Right: By integrating physically based shaders into DVR, a wide spectrum of materials, for instance gold, can be simulated.

Ritschel et al. [21] combine a form of ambient occlusion represented as spherical harmonics (SH), which they call the visibility function, with a DVR approach, relating the emission at each point to both its density and the interaction between the incoming light, from a single source, and the direction-dependent visibility function. Lindemann et al. [22] extend the work of Ritschel et al.[21] with a SH representation of the incident direct and indirect lighting that integrates chromatic attenuation as well as a local approximation of subsurface scattering. They claim to support arbitrary area light sources, but from the paper it is not clear how these are defined. In the work of Kronander et al. [23] lighting, as well as visibility are encoded in the spherical harmonics. Their method supports directional, point and environment lights. In contrast to our DVR framework method, this type of rendering requires extra storage, sensitive to the number of SH coefficients used.

Schott et al. [24] introduced Directional Occlusion, inspired by the AO algorithm. Their method is limited to a light source that has to coincide with the camera. This restriction was later partially removed by allowing the user to place a light within a hemisphere, oriented towards the camera, with the introduction of a multi directional occlusion model by Solteszova et al. [25]. Ropinski et al. enhanced their GPU raycasting framework with simulated scattering and shadowing [2]. The illumination volume was generated with slice-based front-to-back chromaticity accumulation, and in a second pass back-to-front scattering accumulation, and could be generated on the fly when the transfer function or light position was updated. All of these methods yield impressive results, do not require significant pre-computation, and run at interactive frame rates. However, all are limited to modelling a single point light source.

**Figure 10:**
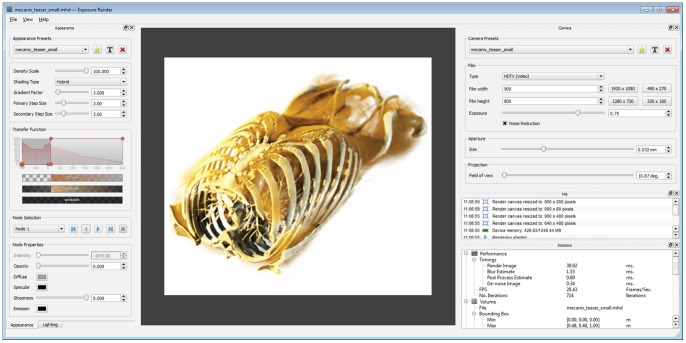
Screenshot of the Exposure Render graphical user interface.

The previously discussed papers yield results that have proven to aid in the perception of shape and depth. The methods render a fairly good approximation of real light interaction with volumes or iso-surfaces. Another class of volume renderers takes a more physically based approach. They typically solve the light transport equation for a volume in a pre-processing step and store the result in an additional buffer. Wyman et al. [26] introduced such a method, in which the direct lighting, shadows and diffuse inter-reflection are captured in an illumination buffer. This buffer is then used to texture iso-surfaces. This method was further developed by Beason et al. [8], introducing translucency and caustics, at the cost of static lighting. Both of these approaches focus on rendering isosurfaces and are not able to do full volumetric rendering.

**Table 1:** Performance.

Data set	Size	Front	Left	Top
Mecanix	512×743×512	35.2	34.1	36.3
Manix	512×460×512	32.7	39.7	42.4
Engine	256×256×128	65.5	53.7	65.7
Bonsai	256×256×257	57.7	66.6	68.1
Artifix	512×347×512	47.3	40.3	50.8

Performance measurements expressed in the number of frame estimates per second for five data sets, rendered at 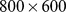 pixels from a front, left and top view respectively. Scenes are lit with an environment light and two additional area lights. Al data sets are encoded in a (16 bit unsigned short) format.

Csébfalvi et al. [27] were the first to apply Monte Carlo integration to volume rendering, with the aim to find a solution to the problem of data sets not fitting on graphics hardware memory. In a pre-processing step, this method generates a point cloud of random samples according to the volume's probability density function. During progressive rendering, from this point cloud, new samples are generated with importance sampling, which are projected back on the image plane and subsequently the pixel intensity is determined by Monte Carlo integration. This method does include gradient vector based lighting effects, for comparison purposes, but does not focus on photo-realistic rendering and ignores occlusion. This work is extended to support real-time modification of the transfer function [27].

**Figure 11:**
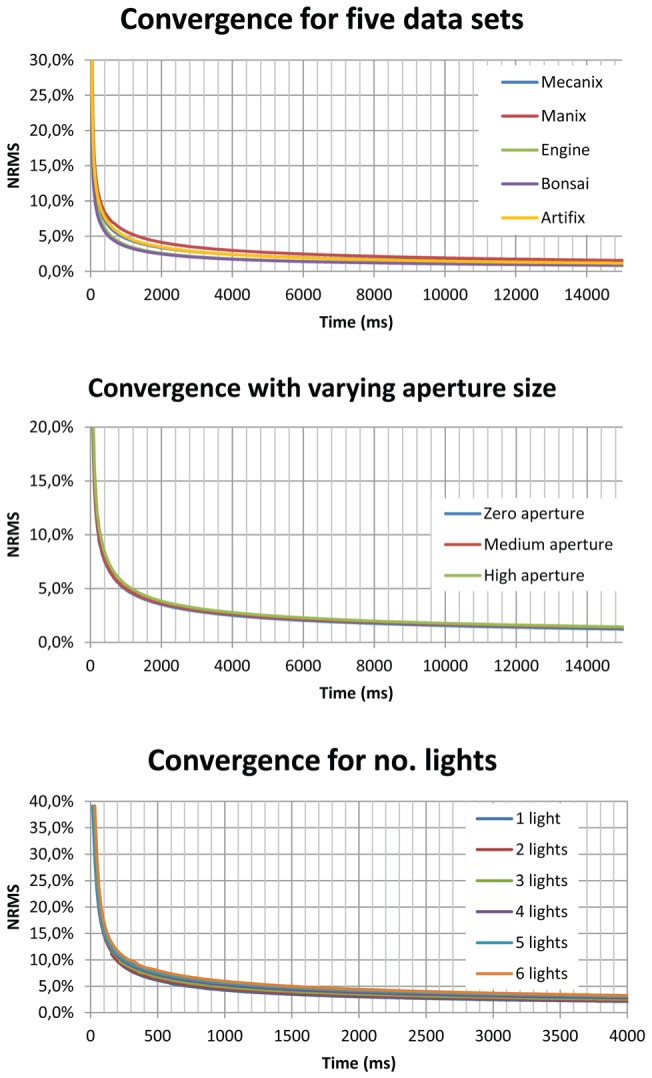
Convergence characteristics. Five typical data sets (top), Manix data set, with constant lighting and shading, subject to infinitely small aperture, medium aperture and large aperture respectively (middle) and the Backpack data set, with constant shading, camera parameters and increasing number of lights (bottom).

Salama et al. [6] presented a GPU framework for Monte Carlo rendering of volumetric data sets. Their work comes closest to ours in the sense that they employ stochastic sampling techniques. However, they render a number of layers using an isosurface in the volume as basis for calculating later scattering. The first pass calculates local illumination on the selected isosurface, the second pass is an AO pass and the final layer, usually rendered with a single pass, models scattering. This is done by starting a transmissive ray at the isosurface, scattered within a Phong lobe, and reflecting this ray from the second isosurface it hits until it exits the volume. The three layers are composited to form the final image. Our DVR framework renders the volume in a unified way, and deliberately does not treat isosurfaces differently.

Schlegel et al.[28] have developed several rendering optimizations based on raycasting. As a result they are able to render ambient occlusion, volumetric shadows and color bleeding in real-time. This method includes soft shadows, AO and color bleeding.

Schott et al. [29] have applied depth-of-field to DVR using a slice based approach. This method employs incremental filtering to blur both iso surfaces and transparent sections. This method creates realistic depth of field effects, at the expense of relatively low refresh rates. Furthermore this method can only be integrated in a slice based renderer such as [24], [25]. Our DVR framework integrates depth-of-field in a unified way using stochastic sampling.

The previous approaches to photo-realistic rendering are often motivated by computational limitations of CPU, GPU and other dedicated hardware. For this reason, most of the work presents compelling, yet approximated, simulation of light transport. We expect that, with more powerful graphics hardware, these approximations will eventually be superseded by physically-based light transport modeling. To our knowledge, the work presented here is the first to demonstrate that interactive, brute force, progressive stochastic rendering for photo-realism in DVR is possible. Our method does not depend on pre-computed quantities or additional volumes and the memory footprint is insensitive to lighting, material, camera and transfer function configurations.

## Methods

At the highest level, our DVR framework continuously estimates the light contribution arriving at the camera film. As this estimate is an approximation, several estimates are needed to form a high quality image. As soon as the transfer function, lighting or camera position are changed, the Monte Carlo integrated estimate buffer is cleared and the MC algorithm starts from the beginning.

Effectively, the image on screen initially appears briefly at a lower quality, but is progressively refined as more frame estimates are integrated. Interaction is instantaneous, as new frame estimates are generated rapidly.

In the following subsections, we describe the DVR framework in more detail.

### Rendering pipeline

Figure 1 depicts the rendering pipeline, which is executed numerous times during progressive refinement. Rendering starts with the stochastic ray-caster, which is discussed in detail in the *Stochastic Raycasting* section. This process yields a High Dynamic Range (HDR) MC estimate of the light arriving at the film plane. This estimate is filtered with a separable Gaussian kernel, with standard deviation 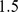 and window 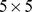 pixels in order to reduce anti-aliasing. Next, Monte Carlo integration is performed by computing the cumulative moving average. The HDR MC estimate is then tone mapped to compress its dynamic range and subsequently gamma corrected. The Low Dynamic Range (LDR) image is then an-isotropically filtered to reduce noise, which is discussed in the *Interactivity* section.

The following sections focus on how we implemented the stochastic ray-casting and the interactivity optimizations.

### Stochastic ray casting

In contrast to standard ray-casting, in which camera rays originate from the camera origin and are cast through pixel centers on the screen, our method implements a thin lens camera model [30], see Figure 2. This model, which incorporates a finite aperture, models a real-world camera. The aperture size controls the depth of field, i.e., the range of distances in which objects appear to be sharp. As a result we are able to easily incorporate depth-of-field effects, which can be used effectively in photo realistic DVR to draw attention to particular regions in an image. Figure 3 shows an example of this phenomenon.

In our implementation, camera rays are constructed by sampling a point on the lens and sampling a perturbed point within the finite area of a pixel on the film plane with stratified sampling. The camera parameters are stored in CUDA constant memory for fast access during ray generation. The rays are intersected with the volume's bounding box, yielding a parametric range 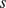. Rays that do not intersect are discarded.

Rays are treated differently in our DVR framework compared to other ray casters, which generally propagate through the volume with Ray Marching (RM). When shadow calculations are required at every sample position along the ray, RM becomes prohibitively slow. To address this problem, we use Woodcock tracking [31], which propagates through the volume with random length steps and yields a single scattering point 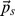. The Woodcock Tracking method determines where a scattering event occurs within the volume by: 1) generating a path length 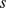 using the maximum extinction coefficient 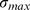 in the volume, 2) accepting the tentative collision point with probability 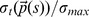 and 3) repeating this step until a collision is accepted, where 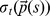 is the extinction coefficient at point 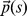 in the volume.

We ultimately implemented Woodcock tracking, because it only yields a single scattering point within the volume, resulting in far fewer shadow computations per estimate. Furthermore, in contrast to ray marching, Woodcock tracking is unbiased. The performance of Woodcock tracking has been further optimized by Kalos et al. [32], but this extension has not been implemented yet in our framework, as this method requires an extinction volume to be recomputed for every transfer function change.

#### Direct lighting

Sampling direct lighting is not as straight-forward as with standard ray casting as our method also includes arbitrarily shaped lights and physically based phase and surface scattering functions, see Figure 2. In order to reduce the sample variance in the Monte Carlo estimator, importance sampling is applied to the phase and surface scattering function for sampling ray directions, and the two samples, obtained by sampling lights and scattering functions, are combined with Multiple Importance Sampling (MIS) as discussed in the work by Veach [33].

In our implementation, the single scattering contribution at scattering point 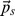 is computed by:

1. Computing the contribution of light that flows along light ray 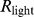 which is formed by connecting the scattering point 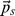 with a stochastically sampled point on a stochastically chosen light source.
2. Sampling the scattering function, yielding the scattering ray 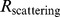. The intersection with a light is used to compute the contribution of light that flows along this ray.
3. Combining the two contributions using MIS by applying the power heuristic.

For the computation of light attenuation we also apply Woodcock tracking. In this case, the location of the scattering point 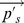, yielded by Woodcock tracking, determines if a light ray is blocked or unblocked. If 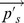 is beyond 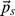, it is unblocked, and vice versa. If the ray is unblocked, the light's exitant radiance 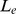 is added. The lights are stored in CUDA constant memory for fast access.

#### Hybrid scattering

The challenge associated with applying MCRT to volumetric data is that there is no explicit boundary surface preset, as with for instance surface meshes. MCRT algorithms that deal with participating media typically are unaware of shading normals, i.e., they use phase functions for computing volumetric scattering.

We present hybrid scattering, a technique that switches stochastically between surface and volumetric scattering based on the local gradient. Hybrid scattering aims to integrate surface and volumetric based scattering into stochastic volume rendering. As a result, volume renderings show detail through specular highlights in areas of well-defined gradient vectors, at the same time including volumetric scattering in areas of ill-defined gradient vectors. As a result the images yielded with this method are not only more vivid, but are also less susceptible to sample variance. The method switches probabilistically between surface and volumetric scattering based on the local voxel gradient magnitude, which is computed on the fly, and an additional weighting parameter controlling the amount of BRDF and volumetric scattering respectively. The gradient vector, used for BRDF calculations is computed on the fly with finite differences.

The method mixes surface and volumetric scattering by stochastically choosing whether a BRDF or phase function is used with the following probability:
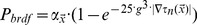
(1)

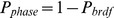
(2)Where 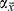 is the opacity at sample position 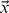, determined by the opacity transfer function. 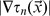 is the normalized gradient magnitude at 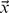 and 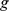 represents the *gradient factor*, a value ranging from [0-1] which controls the amount of surface/volumetric scattering. Hybrid scattering is integrated into our DVR framework by drawing a random number 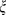 from a uniform distribution and comparing it to the BRDF probability 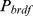:


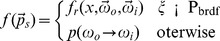
(3)

In our implementation we use an isotropic phase function for volumetric scattering, but other anisotropic phase functions such as Schlick's phase function [34], could be used as well. For surface scattering we implemented the Fresnel blend shading model as described by Ashikmin et al. [35]. It models a diffuse surface with a glossy surface overlaid in a physically plausible way, and is reciprocal and energy conserving, unlike the conventional phong shading in standard ray casters. The fresnel blend BRDF blends between diffuse and glossy reflection by incorporating Schlick's approximation to Fresnel reflectance. The diffuse part is a straightforward Lambert shader, and the glossy part of the Fresnel blend is defined by a microfacet Blinn shader, developed by Blinn [36]. Furthermore, the BRDF is controlled by relatively simple parameters (e.g., diffuse refection, specular reflection and the Blinn exponent, which controls the size of the highlights). We implemented a one dimensional transfer function with the following channels: opacity, emission, diffuse and specular color, specular roughness and index of reflection. Each channel of the transfer function is stored in CUDA texture memory.

Though hybrid scattering is primarily designed to mix surface and volumetric scattering it can also be applied to data sets with lower signal to noise ratio by adjusting the gradient factor.

### Interactivity

MCRT algorithms estimate the incident light on the camera film plane by sampling 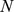 light transport paths and computing the estimate with:
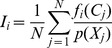



The quality of this estimate is measured with variance, which is the square root of the expected error. As the number of samples 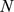 increases, the expected error decreases with 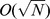. The first few iterations of the MCRT algorithm yield high sample variance estimates, which results in images with high frequency noise, see Figure 4. This noise is an undesirable side effect of MCRT and should be removed as soon as possible, in order to warrant interactivity. In the case of interactive volume rendering it is important that the image quality is high also during interaction. That is, the quality should be such that the user can still discern shape and important details. However, since every interaction triggers a completely restart of the MC algorithm unacceptable high frequency noise is induced.

In order to solve this problem we apply an anisotropic noise reduction filter to the final LDR MC estimate at every iteration, where the amount of noise reduction is dictated by the mean running sample variance of the LDR MC estimate. Previous work, aimed at suppressing noise from MC simulation of light transport has shown impressive results [37]–[40]. However, the performance of these algorithms is not real-time and thus not suitable for our purpose.

For our experiments, we implemented the *K Nearest Neighbor* (KNN) noise reduction filter. The KNN filter is a complex Gaussian blur filter in which the pixel weights are determined by the color similarity of neighboring pixels [41]. The filter runs efficiently on CUDA enabled hardware, and is easy controlled. In our implementation we use a window size of 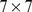 pixels. The *lerpC* parameter of the KNN filter blends between reconstructed and original pixels, and is in our implementation controlled by the mean running sample variance. As the sample variance decreases, so does the influence of the KNN filter. The mean sample variance of the LDR estimate is computed with a numerically stable algorithm, as described in [42]. All other parameters of the filter remain constant during rendering, for more details concerning the KNN filter configuration we refer to our open source implementation.

Figure 5 depicts the development of the LDR MC estimate over time with our noise reduction strategy applied. Note that especially in the first iteration, the noise is greatly reduced, at the expense of slightly increased blurring.

## Results

In this section, we show example renderings generated by our framework, briefly discuss its implementation, and present a number of performance benchmarks.

Example renderings are shown in figures 3, 6, 7, 8 and 9. More example renderings, the implementation itself and demo movies can be found on the Exposure Render website (http://code.google.com/p/exposure-render/).

### Implementation

The techniques discussed in this work are implemented Exposure Render, a complete interactive DVR framework (see Figure 10), which has been made publicly available through a Google Code project: http://code.google.com/p/exposure-render/. The program runs on CUDA enabled graphics hardware, is written in C/C++, contains a full graphical user interface using Qt, and is shipped with sample volume data, transfer function, lighting and camera presets.

At the time of this writing, the software has been downloaded more than a thousand times and it has been reported on by a number of popular graphics community websites.

### Benchmarks

All experiments were performed on a PC with an NVIDIA GeForce GTX 470, with 877 MB of graphics memory, and an Intel ®Core™ i7 CPU 920 with 12GB of RAM. In order to determine the performance of our method we have subjected our renderer to several benchmarks. First, we document the average number of iterations per second for various datasets and viewpoints, see Table 1. We also investigate the convergence characteristics of the DVR framework in second part of our benchmark.

We measured the average number of estimates that our renderer generates per second for a film resolution of 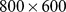 pixels for five typical data sets from three different camera angles. Table 1 shows these results.

Unlike frame-based DVR, our method renders progressively. Convergence is therefore a good performance metric. We benchmark the convergence for five different data sets, varying aperture size and increasing number of lights. This gives a good indication of how robust our DVR framework is with respect to various effects.

We measured convergence by calculating the normalized root mean squared (NRMS), the error between the running MC estimate and a fully converged MC estimate. Figure 11 shows the results of these tests. From this, it can be seen that the image converges quite rapidly and that 10% NRMS is reached in a fraction of a second for all datasets.

Based on these benchmarks, we see that the renderer converges quite rapidly and is robust to different datasets, varying depth-of-field and increasing number of light sources. Importantly, the visualization remains interactive due to the progressive updates, yielding a high-quality volume rendering.

## Discussion

In this paper we introduce a new DVR framework that integrates physically-based lighting in order to achieve photo realistic volume rendering images. We show that a number of effects, which are otherwise hard to obtain, such as realistic shadows, depth-of-field and realistic scattering, can be modeled in a unified way using our framework. Furthermore, we demonstrate that with the necessary optimizations, the brute-force ray-tracing of volumetric data using single scattering and efficient shadow sampling can be done interactively on commodity graphics hardware.

This combination enables physically-based volumetric shadows, any number of arbitrarily positioned, shaped and textured area lights and finally the simulation of a real-world camera, including its lens and aperture. With this setup, we are able to reproduce complex lighting setups that are currently used in photography studios, which adds an extra dimension of expressiveness to our volume visualizations.

In addition to the fact that photo-realistic volume renderings tend to be aesthetically more pleasing, it has been shown that realistic lighting contributes to 3D understanding and can improve depth-related task performance [1]. With this work and the implementation that we have made available, we hope to contribute to the uptake of realistic illumination in interactive direct volume rendering applications. Although the rendered images are progressively refined, the refinement is already quite rapid on mid-level consumer graphics hardware. We expect that either by combining our technique with traditional GPU raycasting techniques in a level-of-detail approach, or through the expected advances in GPU hardware the coming months, the increased realism our work enables will find its use wherever high-quality interactive DVR is currently in place. Examples include radiological diagnosis of complicated 3D pathology, surgical planning, anatomy education and doctor-patient communication.

With this work, one of our goals was to investigate whether graphics hardware has become fast enough to enable the interactive simulation of physically-based light transport. This is part of a broader question on whether direct volume ray-tracing might soon replace direct volume rendering as the interactive volume visualization method of choice. The photo-realistic renderings our framework is able to generate, the added expressiveness and the measured and perceived interactivity of the visualizations, combined with the current trends in graphics hardware development, lead us to answer both questions positively.
