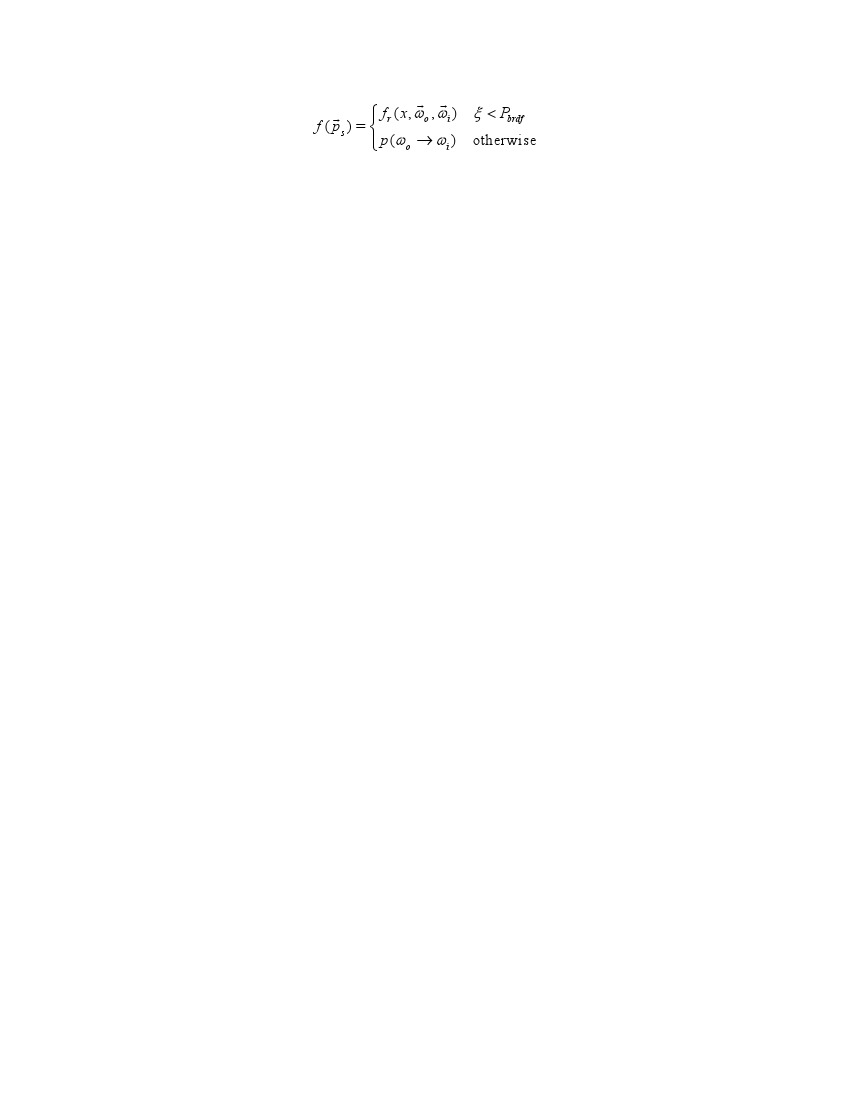# Correction: Exposure Render: An Interactive Photo-Realistic Volume Rendering Framework

**DOI:** 10.1371/annotation/23e9f360-dbf5-434e-9a5a-a78172c6cba8

**Published:** 2013-04-22

**Authors:** Thomas Kroes, Frits H. Post, Charl P. Botha

There was an error in equation 3). The correct equation can be viewed here: